# Supplementary material for: Dopamine mediates life-history responses to food abundance in Daphnia
Source: Proc Biol Sci. 2020 Jul 1;287(1930):20201069. doi: 10.1098/rspb.2020.1069 (PMC7423461; doi:10.1098/rspb.2020.1069)
Supplement: Supporting information [file rspb20201069supp1.doc]

**Electronic supplementary material of the paper**

Dopamine mediates life history responses to food abundance in *Daphnia*

Semona Issa, Marlène Gamelon, Tomasz Maciej Ciesielski, Kristine Vike-Jonas, Alexandros G. Asimakopoulos, Veerle L. B. Jaspers, Sigurd Einum


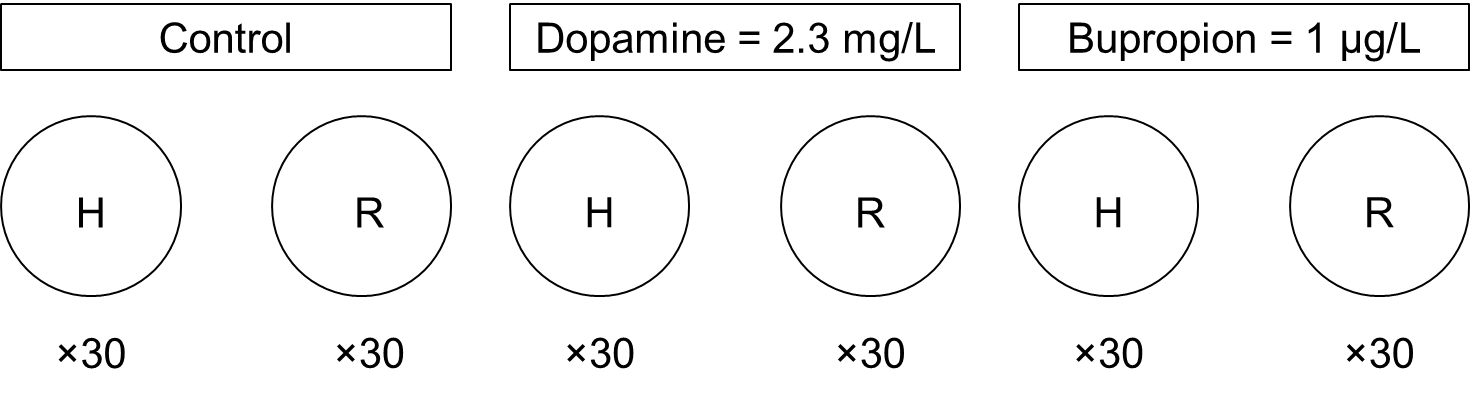


**Fig. A1.** Schematic diagram of the experimental design. Thirty replicates per food ration for the control, dopamine and bupropion treatments (x30). “H” and “R” refer to high and restricted food ration, respectively.

**Bupropion pilot study**

A pilot study was performed to determine appropriate sublethal bupropion test concentrations for *Daphnia magna*. Three bupropion (bupropion hydrochloride (Sigma-Aldrich, St. Louis, MO, USA)) concentrations (1 µg/L, 10 µg/L and 100 µg/L) and a control (0 µg/L bupropion) were applied, with 30 replicates for each of the four treatments. For each treatment, 30 juvenile females of clone 47 were kept individually in 15 mL glass tubes at 20 °C in a modified “Aachener Daphnien Medium” (ADaM). The medium was renewed three times a week during the experimental period, and the animals were maintained until maturity under long photoperiods (16h L: 8h D) and fed with Shellfish Diet 1800® three times a week at a final concentration of 2 × 10^5^ cells/mL. Age and dry mass at maturation were measured at the end of the experiment. In addition, the mortality in each treatment was recorded. Based on our findings (Figures A2 and A3), we chose to use a bupropion concentration of 1 µg/L as this concentration induced significant changes in dry mass and age at maturation without having mortality effects.


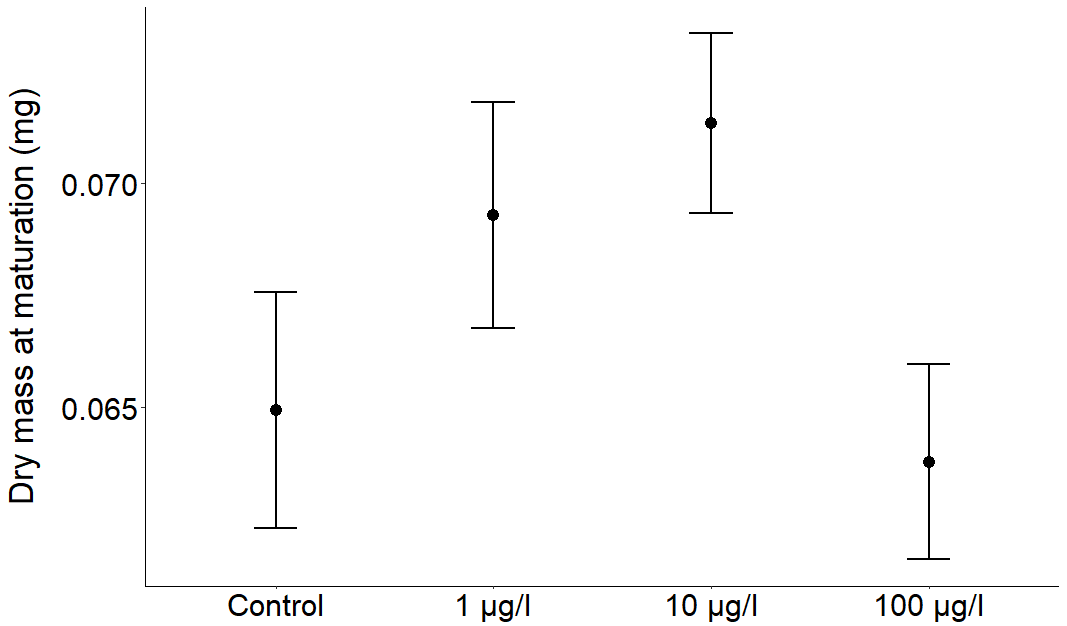


**Fig. A2.** *Daphnia* dry mass at maturation (mg) in response to growth medium bupropion concentrations (mean ± SE).


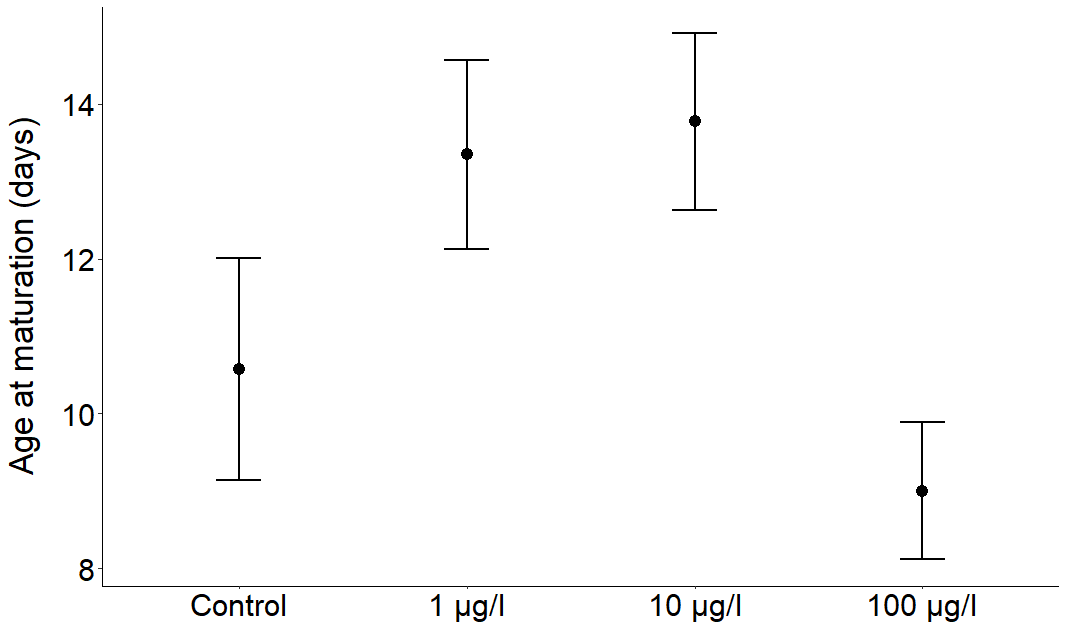


**Fig. A3.** *Daphnia* age at maturation (days) in response to growth medium bupropion concentrations (mean ± SE).


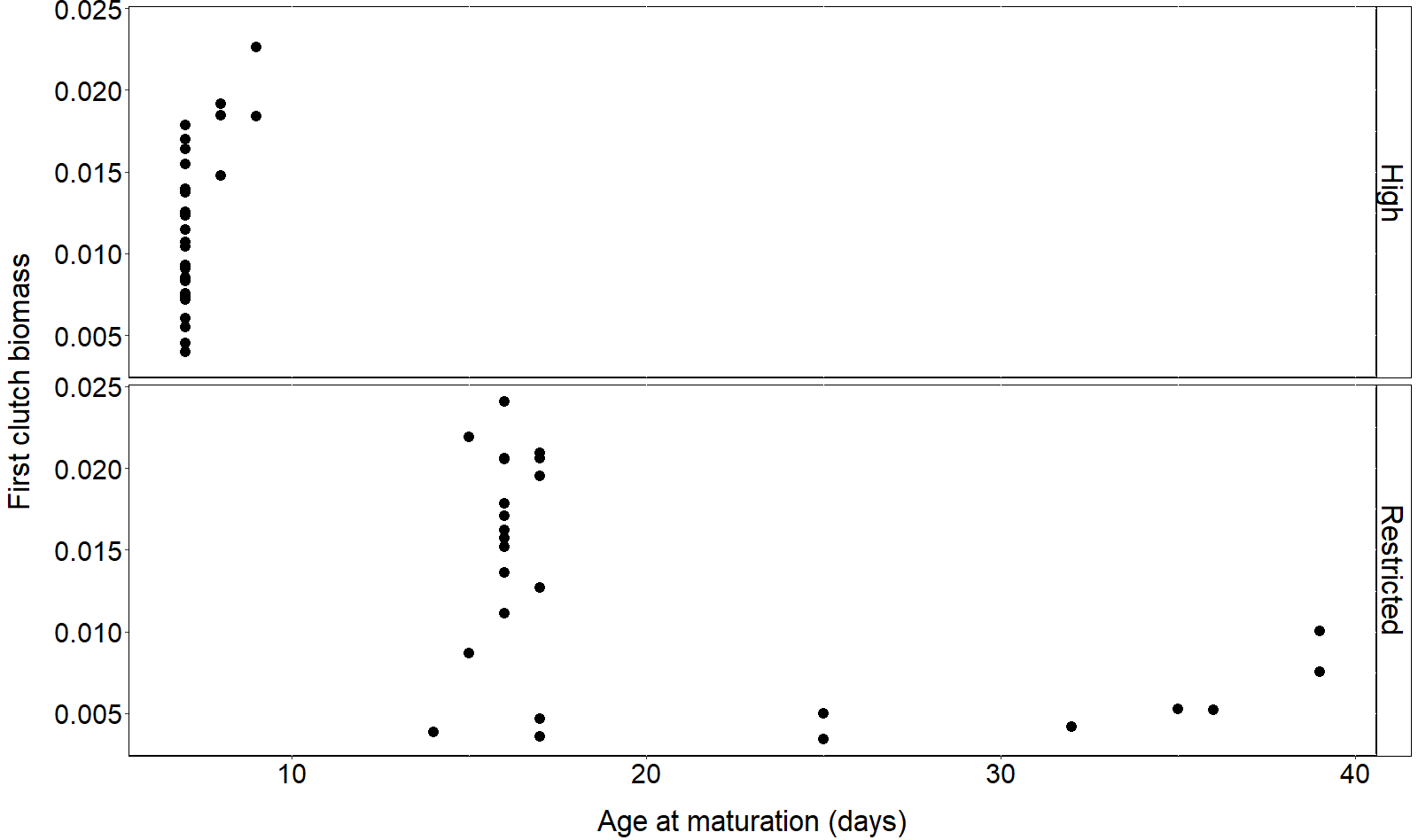


**Fig. A4.** First clutch biomass in response to *Daphnia* age at maturation (days) at restricted and high food rations in the bupropion treatment. The negative effect of age at maturation on biomass is significantly reduced at high food ration.

**UPLC-MS/MS determination of bupropion and dopamine**

The analysis of the samples was performed with two complementary sample preparation protocols to cover all concentration ranges: (1) dilute-and-shoot (Method A); and (2) liquid-liquid extraction (LLE; Method B). In both protocols, all reagent blanks and samples were spiked with a known amount of internal standard (10 ng benzotriazole; IS) prior to initiation of sample preparation. Benzotriazole (≥98%) was purchased from Sigma-Aldrich (Steinheim, Germany). All samples were analysed first with the dilute-and-shoot method, where samples were diluted by a factor of 2, and thereafter analysed by the LLE method, where samples were preconcentrated by a factor of 2. The detect values with method A were further confirmed with method B, while the non-detect values with method A were either detected with method B (due to the preconcentration) or remained as non-detects.

In the dilute-and-shoot method, a volume of 500 μL was transferred in an auto-sampler vial (for UPLC–MS/MS analysis), spiked with IS, and 500 μL of methanol were added reaching a total sample volume of 1 mL. Thereafter, the sample was directly injected to the UPLC–MS/MS system. In the LLE method, a volume of 1 mL of sample was transferred into a 15 mL polypropylene (PP) tube, spiked with IS, 300 μL of 1.0 M ammonium acetate_(aq.)_ were added in the samples, and thereafter, the samples were extracted 3 times with 3 mL of ethyl acetate each time (3 × 3). For each successive extraction, the mixture was shaken in an oscillator shaker for 30 min and then centrifuged. The supernatants were combined, and 2 mL of ultrapure water were added. The mixture was centrifuged again, and the supernatant was transferred into a PP tube and concentrated to near-dryness under a gentle nitrogen stream. Finally, 500 μL of MeOH: ultrapure water (1:1 v/v) was added, vortex mixed and transferred into an auto-sampler vial for UPLC–MS/MS analysis.

The chromatographic separation was carried out using an Acquity UPLC I-Class system (Waters, Milford, U.S.) coupled to a triple quadrupole mass analyser (QqQ; Xevo TQ-S) with a ZSpray ESI ion source (Waters, Milford, U.S.). The used LC column was an Atlantis T3 (150 × 2.1 mm, 3 µm) connected to a Phenomenex C18 guard column (2.1 × 2.0 mm). The injection volume was 2 μL and the column temperature was set at 30 °C. The chromatographic separation was carried out using a gradient elution program with an aquatic (ultrapure water with 0.1% v/v formic acid; A) and an organic phase (methanol with 0.1% v/v formic acid; B) as binary mobile phase at a flow rate of 0.3 mL/min. The gradient elution started at 50% (v/v) A, decreased to 0% A within 3.0 min (3.0^rd^ min), and then reverted to 50% A that was held until the 5.0^th^ min, for a total run time of 5.0 min. The retention times were 1.2 and 2.0 min for dopamine and bupropion, respectively. The electrospray ionisation (ESI) was applied at a potential of +2.5 kV. The cone and source offset voltages were set at 20 and 45V, respectively. The desolvation and cone gas flow rates were set at 800 and 150 L/hr, respectively. The collision gas flow was set at 0.15 mL/min, while the nebuliser gas pressure was set at 87 psi. The source and desolvation temperatures were set at 150 and 350 °C, respectively. The precursor–product ions (transitions), the collision energies and the cone voltage values that were set in the ESI method are presented in Table A1. The instrumental limits of detection (LODs) were calculated for each target analyte as 3 times the signal from the baseline noise (S/N ratio) and were 0.01 and 0.1 ng/mL for bupropion and dopamine, respectively. Quantification of the target drugs was accomplished based on the internal standard method and with matrix-matched standard addition calibration standards prepared by spiking target analytes into the specified matrices prior to extraction [1].

**Table A1.** SRM transitions, collision energies and cone voltage values for UPLC-MS/MS analysis.

| **Derivative** | **Transition 1**  **(T1; Quantitation ion)** | **Transition 2**  **(T2; confirmation ion)** | **Collision energies (V)**  **T1; T2** | **Cone voltage values (V)** |
| --- | --- | --- | --- | --- |
| Dopamine | 154 > 137 | 154 > 91 | 10; 20 | 16 |
| Bupropion | 240 > 184 | 240 > 131 | 12; 26 | 4 |
| Benzotriazole (IS) | 120 > 65 | 120 > 92 | 16; 14 | 28 |

**Table A2.** Exposure and water quality variables averaged over the entire experiment for all replicates. Conductivity, dissolved oxygen, pH and dopamine and bupropion concentrations are summarized by factors treatment (control versus dopamine and bupropion). Values are given as mean ± SE. Means with the same letter are not significantly different from each other (based on Tukey’s post hoc test using an alpha value of 0.05).

|  | **Treatment** | | |
| --- | --- | --- | --- |
|  | Control | Dopamine | Bupropion |
| **Dopamine (mg/L)** | 0.0 ± 0.0 | 0.29 ± 0.2 | 0.0 ± 0.0 |
| **Bupropion (μg/L)** | 0.0 ± 0.0 | 0.0 ± 0.0 | 0.096 ± 0.04 |
| **Conductivity (mS/cm)** | - 1. ± 0.3   a | - 1. ± 0.3   a | - 1. ± 0.3   a |
| **Dissolved oxygen (mg/L)** | 9.0 ± 0.05  a | 9.0 ± 0.05  a | 9.0 ± 0.05  a |
| **pH** | 8.3 ± 0.1  a | 8.2 ± 0.09  a | 8.3 ± 0.1  a |

**Table A3.** Model selection using AICc and quasi-AICc (QAICc) of candidate models for testing effects of treatment (control versus dopamine and bupropion) and food ration (high versus restricted) on somatic growth rate (SGR), age at maturation, age at second reproduction, dry mass (DM) at maturation, 1^st^ and 2^nd^ clutch size, 1^st^ and 2^nd^ clutch offspring DM, longevity; and pH, conductivity and dissolved oxygen in the medium. Models were sorted by ΔAICc and ΔQAICc. The best random effect structure was first determined with REML on models that included all listed fixed effects. Fixed effects were then compared with ML using the best random effect structure. K is the number of parameters estimated. The least complex model within 2 ΔAICc (and ΔQAICc) is bolded. vI refers to the varIdent function.

| **Response variable** | **Model** | **K** | **AICc** | **∆AICc** | **wAICc** |
| --- | --- | --- | --- | --- | --- |
|  |  |  |  |  |  |
| **SGR** |  |  |  |  |  |
| Fixed effects | **SGR ~ Food: Treatment** | 8 | -735.80 | 0.00 | 1.00 |
|  | SGR ~ Food | 4 | -695.10 | 40.71 | 0.00 |
|  | SGR ~ Food + Treatment | 6 | -694.60 | 41.13 | 0.00 |
|  | SGR ~ 1 | 3 | -427.10 | 308.64 | 0.00 |
|  | SGR ~ Treatment | 5 | -423.50 | 312.31 | 0.00 |
| Random effects | **vI (Food)** | 8 | -683.40 | 0.00 | 1.00 |
|  | vI (Treatment) | 9 | -657.50 | 25.84 | 0.00 |
|  |  |  |  |  |  |
| **Age at maturation**  **(days)** |  | **K** | **QAICc** | **∆QAICc** | **wQAICc** |
| Fixed effects | **Age _maturation_ ~ Food: Treatment** | 6 | 713.38 | 0.00 | 1.00 |
|  | Age _maturation_ ~ Food + Treatment | 4 | 745.94 | 32.56 | 0.00 |
|  | Age _maturation_ ~ Food | 2 | 855.38 | 142.00 | 0.00 |
|  | Age _maturation_ ~ Treatment | 3 | 1346.85 | 633.47 | 0.00 |
|  | Age _maturation_ ~ 1 | 1 | 1446.15 | 732.77 | 0.00 |
|  |  |  |  |  |  |
| **DM at maturation (mg)** |  | **K** | **AICc** | **∆AICc** | **wAICc** |
| Fixed effects | **D**M **_maturation_ ~ Food: Treatment** | 8 | -1050.80 | 0.00 | 1.00 |
|  | DM _maturation_ ~ Food + Treatment | 6 | -1030.10 | 20.62 | 0.00 |
|  | DM _maturation_ ~ Food | 4 | -1024.60 | 26.13 | 0.00 |
|  | DM _maturation_ ~ Treatment | 5 | -942.00 | 108.75 | 0.00 |
|  | DM _maturation_ ~ 1 | 3 | -933.50 | 117.23 | 0.00 |
| Random effects | **vI (Food)** | 8 | -987.30 | 0.00 | 1.00 |
|  | vI (Treatment) | 9 | -963.00 | 24.30 | 0.00 |
|  |  |  |  |  |  |
| **1^st^ clutch size** |  | **K** | **AICc** | **∆AICc** | **wAICc** |
| Fixed effects | 1^st^ clutch size ~ Food: Treatment | 6 | 654.60 | 0.00 | 0.51 |
|  | **1^st^ clutch size** **~ Food + Treatment** | 4 | 654.70 | 0.09 | 0.49 |
|  | 1^st^ clutch size ~ Food | 2 | 667.40 | 12.87 | 0.001 |
|  | 1^st^ clutch size ~ Treatment | 3 | 707.20 | 52.62 | 0.00 |
|  | 1^st^ clutch size ~ 1 | 1 | 719.70 | 65.13 | 0.00 |
|  |  |  |  |  |  |
| **Age at 2^nd^ reproduction**  **(days)** |  | **K** | **AICc** | **∆AICc** | **wAICc** |
| Fixed effects | **Age _2_^nd^ _reproduction_ ~ Food: Treatment** | 6 | 943.80 | 0.00 | 1.00 |
|  | Age _2_^nd^ _reproduction_ ~ Food + Treatment | 4 | 975.40 | 31.54 | 0.00 |
|  | Age _2_^nd^ _reproduction_ ~ Food | 2 | 1062.40 | 118.55 | 0.00 |
|  | Age _2_^nd^ _reproduction_ ~ Treatment | 3 | 2137.10 | 1193.22 | 0.00 |
|  | Age _2_^nd^ _reproduction_ ~ 1 | 1 | 2200.90 | 1257.04 | 0.00 |
|  |  |  |  |  |  |
| **2^nd^ clutch size** |  | **K** | **AICc** | **∆AICc** | **wAICc** |
| Fixed effects | **2^nd^ clutch size ~ Food: Treatment** | 6 | 622.90 | 0.00 | 0.95 |
|  | 2^nd^ clutch size ~ Food | 2 | 629.80 | 6.86 | 0.03 |
|  | 2^nd^ clutch size ~ Food + Treatment | 4 | 631.20 | 8.25 | 0.01 |
|  | 2^nd^ clutch size ~ 1 | 1 | 777.40 | 154.51 | 0.00 |
|  | 2^nd^ clutch size ~ Treatment | 3 | 779.70 | 156.78 | 0.00 |
|  |  |  |  |  |  |
| **1^st^ clutch offspring DM**  **(mg)** |  | **K** | **AICc** | **∆AICc** | **wAICc** |
| Fixed effects | **1^st^ clutch offspring DM ~ Food + Treatment** | 7 | -4876.30 | 0.00 | 0.79 |
|  | 1^st^ clutch offspring DM ~ Food: Treatment | 9 | -4873.60 | 2.73 | 0.20 |
|  | 1^st^ clutch offspring DM ~ Food | 5 | -4848.60 | 27.76 | 0.00 |
|  | 1^st^ clutch offspring DM ~ Treatment | 6 | -4764.50 | 111.83 | 0.00 |
|  | 1^st^ clutch offspring DM ~ 1 | 4 | -4752.40 | 123.95 | 0.00 |
| Random effects | **vI (Food) + (1 | Replicate)** | 9 | -4778.60 | 0.00 | 0.78 |
|  | vI (Treatment) + (1 \| Replicate) | 10 | -4775.80 | 2.77 | 0.19 |
|  | (1 \| Replicate) | 8 | -4771.50 | 7.02 | 0.02 |
|  | vI (Food) | 8 | -4568.80 | 209.73 | 0.00 |
|  | vI (Treatment) | 9 | -4555.90 | 222.66 | 0.00 |
|  |  |  |  |  |  |
| **Offspring DM 2^nd^ clutch (mg)** |  | **K** | **AICc** | **∆AICc** | **wAICc** |
| Fixed effects | **2^nd^ clutch offspring DM ~ Food + Treatment** | 6 | -1919.60 | 0.00 | 0.83 |
|  | 2^nd^ clutch offspring DM ~ Food: Treatment | 8 | -1915.60 | 3.97 | 0.11 |
|  | 2^nd^ clutch offspring DM ~ Treatment | 5 | -1912.50 | 7.10 | 0.02 |
|  | 2^nd^ clutch offspring DM ~ Food | 4 | -1912.40 | 7.23 | 0.02 |
|  | 2^nd^ clutch offspring DM ~ 1 | 3 | -1910.80 | 8.84 | 0.01 |
| Random effects | **(1 | Replicate)** | 8 | -1830.00 | 0.00 | 0.59 |
|  | vI (Food) + (1 \| Replicate) | 9 | -1828.80 | 1.24 | 0.31 |
|  | vI (Treatment) + (1 \| Replicate) | 10 | -1826.40 | 3.61 | 0.10 |
|  | vI (Treatment) | 9 | -1765.60 | 64.40 | 0.00 |
|  | vI (Food) | 8 | -1757.70 | 72.32 | 0.00 |
|  |  |  |  |  |  |
| **Longevity (days)** |  | **K** | **QAICc** | **∆QAICc** | **wQAICc** |
| Fixed effects | **Longevity ~ Food: Treatment** | 6 | 365.83 | 0.00 | 1.00 |
|  | Longevity ~ Treatment | 3 | 393.53 | 27.70 | 0.00 |
|  | Longevity ~ Food + Treatment | 4 | 395.47 | 29.65 | 0.00 |
|  | Longevity ~ 1 | 1 | 396.45 | 30.63 | 0.00 |
|  | Longevity ~ Food | 2 | 398.36 | 32.53 | 0.00 |
|  |  |  |  |  |  |
| **pH** |  | **K** | **AICc** | **∆AICc** | **wAICc** |
| Fixed effects | **pH ~ 1** | 2 | 11.00 | 0.00 | 0.90 |
|  | pH ~ Treatment | 4 | 15.50 | 4.43 | 0.10 |
|  |  |  |  |  |  |
| **Conductivity (mS/cm)** |  | **K** | **AICc** | **∆AICc** | **wAICc** |
| Fixed effects | **Conductivity ~ 1** | 2 | 67.60 | 0.00 | 0.93 |
|  | Conductivity ~ Treatment | 4 | 73.00 | 5.32 | 0.07 |
|  |  |  |  |  |  |
| **Dissolved oxygen (mg/L)** |  | **K** | **AICc** | **∆AICc** | **wAICc** |
| Fixed effects | **Dissolved oxygen ~ 1** | 2 | -25.60 | 0.00 | 0.90 |
|  | Dissolved oxygen ~ Treatment | 4 | -21.30 | 4.32 | 0.10 |

**Table A4.** Summary statistics of fitted final models.

| **Response variable** | **Final model** | **Parameter** | **Estimate ± SE** |
| --- | --- | --- | --- |
| **SGR** | SGR ~ Food: Treatment +  vI (Food) | Intercept | 0.37 ± 0.004 |
|  |  | Dopamine treatment | -0.003 ± 0.005 |
|  |  | Bupropion treatment | -0.004 ± 0.005 |
|  |  | Restricted food | -0.27 ± 0.008 |
|  |  | Restricted food: Dopamine treatment | 0.07 ± 0.01 |
|  |  | Restricted food: Bupropion treatment | 0.06 ± 0.01 |
|  |  |  |  |
| **Age at maturation (days)** | Age _maturation_ ~  Food: Treatment | Intercept | 1.96 ± 0.08 |
|  |  | Dopamine treatment | 0.04 ± 0.12 |
|  |  | Bupropion treatment | 0.01 ± 0.11 |
|  |  | Restricted food | 1.60 ± 0.09 |
|  |  | Restricted food: Dopamine treatment | -0.76 ± 0.13 |
|  |  | Restricted food: Bupropion treatment | -0.57 ± 0.13 |
|  |  |  |  |
| **DM at maturation (mg)** | DM _maturation_ ~  Food: Treatment+ vI (Food) | Intercept | 0.07 ± 0.001 |
|  |  | Dopamine treatment | 0.007 ± 0.002 |
|  |  | Bupropion treatment | -0.0004 ± 0.002 |
|  |  | Restricted food | 0.036 ± 0.003 |
|  |  | Restricted food: Dopamine treatment | -0.02 ± 0.004 |
|  |  | Restricted food: Bupropion treatment | -0.01 ± 0.004 |
|  |  |  |  |
| **1^st^ clutch size** | 1^st^ clutch size ~ Food + Treatment | Intercept | 1.43 ± 0.08 |
|  |  | Dopamine treatment | 0.31 ± 0.10 |
|  |  | Bupropion treatment | 0.36 ± 0.09 |
|  |  | Restricted food | -0.57 ± 0.08 |
|  |  |  |  |
| **Age at 2^nd^ reproduction (days)** | Age _2_^nd^ _reproduction_ ~  Food: Treatment | Intercept | 2.31 ± 0.06 |
|  |  | Dopamine treatment | 0.03 ± 0.08 |
|  |  | Bupropion treatment | -0.02 ± 0.08 |
|  |  | Restricted food | 1.45 ± 0.07 |
|  |  | Restricted food: Dopamine treatment | -0.58 ± 0.10 |
|  |  | Restricted food: Bupropion treatment | -0.14 ± 0.09 |
|  |  |  |  |
| **2^nd^ clutch size** | 2^nd^ clutch size ~ Food: Treatment | Intercept | 1.97 ± 0.07 |
|  |  | Dopamine treatment | -0.14 ± 0.10 |
|  |  | Bupropion treatment | -0.13 ± 0.10 |
|  |  | Restricted food | -1.30 ± 0.16 |
|  |  | Restricted food: Dopamine treatment | 0.68 ± 0.21 |
|  |  | Restricted food: Bupropion treatment | 0.11 ± 0.23 |
|  |  |  |  |
| **Offspring DM 1^st^ clutch (mg)** | 1^st^ clutch offspring DM ~ Food + Treatment +  vI (Food) + (1 | Replicate) | Intercept | 0.003 ± 0.0001 |
|  |  | Dopamine treatment | -0.0004 ± 0.0001 |
|  |  | Bupropion treatment | -0.0009 ± 0.0001 |
|  |  | Restricted food | 0.001 ± 0.0001 |
|  |  |  |  |
| **Offspring DM 2^nd^ clutch (mg)** | 2^nd^ clutch offspring DM ~ Food + Treatment +  (1 | Replicate) | Intercept | 0.004 ± 0.0003 |
|  |  | Dopamine treatment | -0.0009 ± 0.0003 |
|  |  | Bupropion treatment | -0.0006 ± 0.0003 |
|  |  | Restricted food | 0.0009 ± 0.0003 |
|  |  |  |  |
| **Longevity (days)** | Longevity ~ Food: Treatment | Intercept | 4.21 ± 0.05 |
|  |  | Dopamine treatment | 0.31 ± 0.07 |
|  |  | Bupropion treatment | 0.07 ± 0.07 |
|  |  | Restricted food | 0.12 ± 0.07 |
|  |  | Restricted food: Dopamine treatment | -0.44 ± 0.10 |
|  |  | Restricted food: Bupropion treatment | 0.10 ± 0.10 |
|  |  |  |  |
| **pH** | pH ~ 1 | Intercept | 8.28 ± 0.05 |
|  |  |  |  |
| **Conductivity (mS/cm)** | Conductivity ~ 1 | Intercept | 1.06 ± 0.15 |
|  |  |  |  |
| **Dissolved oxygen (mg/L)** | Dissolved oxygen ~ 1 | Intercept | 8.99 ± 0.03 |

**Table A5. Mean trait responses to high and restricted food rations are compared between the control, and the dopamine and bupropion treatments. Statistically significant differences from the control group are reported as average percentage changes. “+” and “─” indicate an increase and a decrease, respectively, in mean trait response compared to the control. Means that are not significantly different from each other are reported as “ns”.**

|  | **High food** | | **Restricted food** | |
| --- | --- | --- | --- | --- |
| **Trait** | Dopamine | Bupropion | Dopamine | Bupropion |
| **Somatic growth rate** | ns | ns | + 72 % | + 57 % |
| **Age at maturation (days)** | ns | ns | ̶ 51 % | ̶ 43 % |
| **Mass at maturation**  **(mg dry mass)** | + 11 % | ns | ̶ 14 % | ̶ 15 % |
| **1^st^ clutch size** | + 32 % | + 26 % | + 48 % | + 86 % |
| **Age at 2^nd^ reproduction (days)** | ns | ns | ̶ 42 % | ̶ 15 % |
| **2^nd^ clutch size** | ns | ns | + 72 % | ns |
| **Offspring mass 1^st^ clutch (mg dry mass)** | ̶ 13 % | ̶ 26 % | ̶ 15 % | ̶ 26 % |
| **Offspring mass 2^nd^ clutch (mg dry mass)** | ̶ 14 % | ̶ 21 % | ̶ 21 % | ̶ 10 % |
| **Longevity (days)** | + 37 % | ns | ns | ns |
| **Intrinsic population growth rate** | ns | ns | + 159 % | + 114 % |

**Table A6.** Mean trait responses to high and restricted food rations in the dopamine group are compared to those in the bupropion group. Statistically significant differences are reported as average percentage changes in the dopamine group compared to the bupropion group. “+” and “─” indicate an increase and a decrease, respectively, in mean trait response under the effect of dopamine compared to that of bupropion. Means that are not significantly different from each other are reported as “ns”.

|  | **High food** | **Restricted food** |
| --- | --- | --- |
| **Trait** | Dopamine vs. Bupropion | Dopamine vs. Bupropion |
| **Somatic growth rate** | ns | ns |
| **Age at maturation (days)** | ns | ns |
| **Mass at maturation**  **(mg dry mass)** | + 12 % | ns |
| **1^st^ clutch size** | ns | ns |
| **Age at 2^nd^ reproduction (days)** | ns | ─ 32 % |
| **2^nd^ clutch size** | ns | + 76 % |
| **Offspring mass 1^st^ clutch (mg dry mass)** | + 17 % | + 16 % |
| **Offspring mass 2^nd^ clutch (mg dry mass)** | ns | ns |
| **Longevity (days)** | + 27 % | ─ 26 % |
| **Intrinsic population growth rate** | ns | ns |

**Table A7.** Coefficient values, standard errors (SE), degrees of freedom (DF), z-scores, P-values (*P*) and standardized path coefficients for each fitted structural equation model.

| **Path** | **Estimate** | **SE** | **DF** | **z value** | ***P*** | **Standardized estimate** |
| --- | --- | --- | --- | --- | --- | --- |
|  |  |  |  |  |  |  |
| **Control** |  |  |  |  |  |  |
| Food ration → Age at maturation | -27.825 | 2.3207 | 54 | -11.9899 | 0 | -0.8526 |
| Food ration → DM at maturation | -0.0014 | 0.0035 | 53 | -0.3885 | 0.6992 | -0.0309 |
| Age at maturation → DM at maturation | 0.0012 | 0.0001 | 53 | 11.6448 | 0 | 0.9266 |
| Age at maturation → First clutch biomass | 0.0003 | 0.0001 | 53 | 3.7021 | 0.0005 | 0.8276 |
| Food ration → First clutch biomass | 0.0108 | 0.0024 | 53 | 4.4973 | 0 | 1.0053 |
| First clutch biomass ~~ DM at maturation | 0.3402 | NA | 56 | 2.6338 | 0.0055 | 0.3402 |
|  |  |  |  |  |  |  |
| **Dopamine** |  |  |  |  |  |  |
| Food ration → Age at maturation | -9.8648 | 0.6987 | 52 | -14.1195 | 0 | -0.8906 |
| Food ration → DM at maturation | 0.0256 | 0.0053 | 51 | 4.8230 | 0 | 0.8516 |
| Age at maturation → DM at maturation | 0.004 | 0.0005 | 51 | 8.3831 | 0 | 1.4802 |
| Age at maturation → First clutch biomass | 0.0014 | 0.0003 | 51 | 4.8265 | 0 | 1.2024 |
| Food ration → First clutch biomass | 0.0162 | 0.0031 | 51 | 5.1612 | 0 | 1.2857 |
| First clutch biomass ~~ DM at maturation | 0.7172 | NA | 54 | 7.3493 | 0 | 0.7172 |
|  |  |  |  |  |  |  |
| **Bupropion** |  |  |  |  |  |  |
|  |  |  |  |  |  |  |
| Food ration → Age at maturation | -13.4038 | 1.5550 | 52 | -8.6198 | 0 | -0.767 |
| Food ration → DM at maturation | -0.0068 | 0.0027 | 51 | -2.5066 | 0.0154 | -0.2398 |
| Age at maturation → DM at maturation | 0.0011 | 0.0002 | 51 | 7.3336 | 0 | 0.7016 |
| Age at maturation → First clutch biomass | -0.0004 | 0.0001 | 50 | -3.5641 | 0.0008 | -0.6487 |
| Food ration → First clutch biomass | -0.0491 | 0.0123 | 50 | -3.9776 | 0.0002 | -4.1701 |
| Food ration: Age at maturation → First clutch biomass | 0.0059 | 0.0017 | 50 | 3.5393 | 0.0009 | 3.6415 |
| First clutch biomass ~~ DM at maturation | 0.2835 | NA | 54 | 2.1114 | 0.0198 | 0.2835 |
|  |  |  |  |  |  |  |

**Table A8. Dataset for maternal traits. Replicate id corresponds to mother id.**

| **Food ration** | **Treatment** | **Size at maturation (mm)** | **Replicate**  **id** | **Age at maturation (days)** | **First**  **clutch size** | **Age at 2^nd^ reproduction (days)** | **Second clutch size** | **Longevity (days)** | **Survival probability till 1st clutch** | **Survival probability till 2nd clutch** |
| --- | --- | --- | --- | --- | --- | --- | --- | --- | --- | --- |
| High | Control | 2.611 | 1 | 8 | 5 | 11 | 3 | 65 | 1 | 1 |
| High | Control | 2.464 | 2 | 7 | 4 | 10 | 7 | 78 | 1 | 1 |
| High | Control | 2.406 | 3 | 7 | 6 | 10 | 8 | 67 | 1 | 1 |
| High | Control | 2.490 | 4 | 7 | 5 | 10 | 6 | 57 | 1 | 1 |
| High | Control | 2.522 | 5 | 7 | 5 | 10 | 10 | NA | 1 | 1 |
| High | Control | 2.468 | 6 | 7 | 5 | 10 | 7 | 60 | 1 | 1 |
| High | Control | 2.517 | 7 | 7 | 4 | 10 | 7 | 55 | 1 | 1 |
| High | Control | 2.419 | 8 | 7 | 2 | 9 | 10 | 57 | 1 | 1 |
| High | Control | 2.695 | 9 | 9 | 7 | 15 | 6 | 57 | 1 | 1 |
| High | Control | 2.533 | 10 | 7 | 5 | 10 | 7 | 72 | 1 | 1 |
| High | Control | 2.439 | 11 | 7 | 3 | 9 | 8 | 60 | 1 | 1 |
| High | Control | 2.600 | 12 | 7 | 4 | 10 | 9 | 62 | 1 | 1 |
| High | Control | 2.444 | 13 | 7 | 3 | 10 | 7 | 57 | 1 | 1 |
| High | Control | 2.432 | 14 | 7 | 3 | 10 | 7 | 62 | 1 | 1 |
| High | Control | 2.557 | 15 | 7 | 5 | 10 | 7 | 96 | 1 | 1 |
| High | Control | 2.494 | 16 | 7 | 4 | 10 | 8 | 102 | 1 | 1 |
| High | Control | 2.462 | 17 | 7 | 3 | 9 | 7 | 63 | 1 | 1 |
| High | Control | 2.452 | 18 | 8 | 4 | 12 | 4 | 57 | 1 | 1 |
| High | Control | 2.502 | 19 | 7 | 4 | 10 | 7 | 58 | 1 | 1 |
| High | Control | 2.601 | 20 | 7 | 4 | 10 | 8 | 108 | 1 | 1 |
| High | Control | 2.520 | 21 | 7 | 4 | 9 | 7 | 100 | 1 | 1 |
| High | Control | 2.545 | 22 | 7 | 5 | 11 | 5 | 65 | 1 | 1 |
| High | Control | 2.571 | 23 | 7 | 4 | 10 | 8 | 67 | 1 | 1 |
| High | Control | 2.496 | 24 | 7 | 5 | 9 | 7 | 47 | 1 | 1 |
| High | Control | 2.687 | 25 | 7 | 5 | 10 | 9 | 55 | 1 | 1 |
| High | Control | 2.579 | 26 | 7 | NA | NA | NA | NA | NA | NA |
| High | Control | 2.576 | 27 | 7 | 4 | 9 | 8 | 58 | 1 | 1 |
| High | Control | 2.627 | 28 | 7 | 7 | 10 | 7 | 62 | 1 | 1 |
| High | Control | 2.585 | 29 | 7 | 6 | 10 | 8 | 76 | 1 | 1 |
| High | Control | 2.436 | 30 | 7 | 4 | 9 | 6 | 57 | 1 | 1 |
| Low | Control | 3.074 | 1 | 31 | 2 | 35 | 1 | 77 | 1 | 1 |
| Low | Control | 2.848 | 2 | 24 | 2 | 32 | 2 | 79 | 1 | 1 |
| Low | Control | 2.622 | 3 | 17 | 1 | 29 | 1 | 159 | 1 | 1 |
| Low | Control | 2.595 | 4 | 17 | 1 | 25 | 2 | 88 | 1 | 1 |
| Low | Control | 2.976 | 5 | 30 | 2 | 39 | 1 | 57 | 1 | 1 |
| Low | Control | 2.938 | 6 | 30 | 1 | 43 | 1 | 88 | 1 | 1 |
| Low | Control | 2.747 | 7 | 18 | 1 | 44 | 2 | 57 | 1 | 1 |
| Low | Control | 2.885 | 8 | 30 | 1 | 41 | NA | 57 | 1 | 1 |
| Low | Control | 2.572 | 9 | 18 | 1 | 37 | 1 | 126 | 1 | 1 |
| Low | Control | 2.570 | 10 | 10 | 2 | 37 | 2 | 57 | 1 | 1 |
| Low | Control | 3.214 | 11 | 50 | 2 | 52 | 3 | 91 | 1 | 1 |
| Low | Control | 3.207 | 12 | 42 | 2 | 46 | 2 | 54 | 1 | 1 |
| Low | Control | 2.892 | 13 | 25 | 1 | 36 | 1 | 58 | 1 | 1 |
| Low | Control | 2.843 | 14 | 29 | 1 | 44 | 2 | 93 | 1 | 1 |
| Low | Control | 3.101 | 15 | 42 | 3 | 50 | 4 | 57 | 1 | 1 |
| Low | Control | 2.982 | 16 | 30 | 3 | 39 | 2 | 98 | 1 | 1 |
| Low | Control | 3.064 | 17 | 42 | 6 | 50 | 3 | 124 | 1 | 1 |
| Low | Control | 2.882 | 18 | 36 | 1 | 40 | 1 | 57 | 1 | 1 |
| Low | Control | NA | 19 | NA | NA | NA | NA | NA | NA | NA |
| Low | Control | 3.025 | 20 | 39 | 3 | 42 | 2 | 114 | 1 | 1 |
| Low | Control | 3.174 | 21 | 50 | 4 | NA | NA | 57 | 1 | 0 |
| Low | Control | 2.983 | 22 | 49 | 3 | 65 | 2 | 69 | 1 | 1 |
| Low | Control | 3.043 | 23 | 44 | 2 | 51 | 2 | 57 | 1 | 1 |
| Low | Control | 3.091 | 24 | 52 | 2 | 56 | 2 | 84 | 1 | 1 |
| Low | Control | 2,831 | 25 | 30 | NA | NA | NA | 57 | 1 | NA |
| Low | Control | 3.112 | 26 | 43 | 1 | 47 | 2 | 57 | 1 | 1 |
| Low | Control | 2.984 | 27 | 44 | 2 | 55 | 4 | 57 | 1 | 1 |
| Low | Control | 3.069 | 28 | 51 | 2 | NA | NA | 57 | 1 | 0 |
| Low | Control | 3.194 | 29 | 51 | 3 | NA | NA | 57 | 1 | 0 |
| Low | Control | 3.120 | 30 | 51 | 3 | NA | NA | 57 | 1 | 0 |
| High | Dopamine | NA | 1 | NA | NA | NA | NA | NA | NA | NA |
| High | Dopamine | 2.712 | 2 | 8 | 7 | 11 | 5 | 93 | 1 | 1 |
| High | Dopamine | NA | 3 | NA | NA | NA | NA | NA | NA | NA |
| High | Dopamine | 2.417 | 4 | 7 | 4 | 9 | 7 | 101 | 1 | 1 |
| High | Dopamine | NA | 5 | NA | NA | NA | NA | NA | NA | NA |
| High | Dopamine | 2.835 | 6 | 9 | 7 | 12 | 5 | 67 | 1 | 1 |
| High | Dopamine | 2.597 | 7 | 8 | 6 | 11 | 5 | 113 | 1 | 1 |
| High | Dopamine | 2.383 | 8 | 7 | 6 | 9 | 5 | 66 | 1 | 1 |
| High | Dopamine | 2.436 | 9 | 7 | 3 | 9 | 8 | 60 | 1 | 1 |
| High | Dopamine | 2.443 | 10 | 7 | 5 | 10 | 7 | 67 | 1 | 1 |
| High | Dopamine | NA | 11 | NA | NA | NA | NA | NA | NA | NA |
| High | Dopamine | 2.722 | 12 | 7 | 8 | 10 | 8 | 97 | 1 | 1 |
| High | Dopamine | 2.657 | 13 | 7 | 6 | 11 | 6 | 109 | 1 | 1 |
| High | Dopamine | 2.729 | 14 | 7 | 9 | 11 | 6 | 97 | 1 | 1 |
| High | Dopamine | 2.597 | 15 | 7 | 5 | 10 | 9 | 134 | 1 | 1 |
| High | Dopamine | 2.600 | 16 | 7 | 4 | 9 | 7 | 86 | 1 | 1 |
| High | Dopamine | 2.704 | 17 | 7 | 7 | 11 | 6 | 118 | 1 | 1 |
| High | Dopamine | 2.882 | 18 | 9 | 6 | 12 | 2 | 82 | 1 | 1 |
| High | Dopamine | 2.602 | 19 | 7 | 6 | 10 | 6 | 57 | 1 | 1 |
| High | Dopamine | 2.665 | 20 | 7 | 5 | 10 | 7 | NA | 1 | 1 |
| High | Dopamine | 2.626 | 21 | 7 | 6 | 11 | 6 | 112 | 1 | 1 |
| High | Dopamine | 2.559 | 22 | 7 | 6 | 10 | 8 | 109 | 1 | 1 |
| High | Dopamine | 2.563 | 23 | 7 | 6 | 10 | 8 | 94 | 1 | 1 |
| High | Dopamine | 2.486 | 24 | 7 | 5 | 9 | 9 | 106 | 1 | 1 |
| High | Dopamine | 2.547 | 25 | 7 | 4 | 9 | 8 | 116 | 1 | 1 |
| High | Dopamine | 2.413 | 26 | 9 | 5 | 12 | 5 | 53 | 1 | 1 |
| High | Dopamine | 2.616 | 27 | 7 | 7 | 10 | 7 | 86 | 1 | 1 |
| High | Dopamine | 2.627 | 28 | 7 | 7 | 10 | 8 | 76 | 1 | 1 |
| High | Dopamine | 2.870 | 29 | 9 | 6 | 12 | 1 | 76 | 1 | 1 |
| High | Dopamine | 2.854 | 30 | 9 | 7 | 12 | 3 | 122 | 1 | 1 |
| Low | Dopamine | 3.162 | 1 | 23 | 8 | 44 | 1 | 58 | 1 | 1 |
| Low | Dopamine | 2.806 | 2 | 17 | 2 | 21 | 1 | 58 | 1 | 1 |
| Low | Dopamine | 3.133 | 3 | 22 | 7 | 52 | 1 | 58 | 1 | 1 |
| Low | Dopamine | 2.612 | 4 | 14 | 2 | 22 | 2 | 96 | 1 | 1 |
| Low | Dopamine | 2.777 | 5 | 15 | 2 | 22 | 7 | 62 | 1 | 1 |
| Low | Dopamine | 2.751 | 6 | 16 | 2 | 21 | 1 | 62 | 1 | 1 |
| Low | Dopamine | 2.756 | 7 | 15 | 2 | 22 | 7 | 58 | 1 | 1 |
| Low | Dopamine | 2.604 | 8 | 14 | 1 | 18 | 1 | 110 | 1 | 1 |
| Low | Dopamine | 2.748 | 9 | 16 | 2 | 25 | 6 | 58 | 1 | 1 |
| Low | Dopamine | 2.691 | 10 | 14 | 2 | 22 | 2 | 62 | 1 | 1 |
| Low | Dopamine | 3.066 | 11 | 22 | 8 | 26 | NA | 115 | 1 | 1 |
| Low | Dopamine | 2.579 | 12 | 15 | 2 | 22 | 5 | 62 | 1 | 1 |
| Low | Dopamine | 2.752 | 13 | 15 | 3 | 22 | 6 | 61 | 1 | 1 |
| Low | Dopamine | 2.863 | 14 | 16 | 2 | 24 | 8 | 61 | 1 | 1 |
| Low | Dopamine | 2.705 | 15 | 15 | 3 | 22 | 5 | 57 | 1 | 1 |
| Low | Dopamine | 3.104 | 16 | 23 | 8 | NA | NA | 57 | 1 | 0 |
| Low | Dopamine | 2.640 | 17 | 15 | 3 | 22 | 6 | 62 | 1 | 1 |
| Low | Dopamine | 2.809 | 18 | 16 | 2 | 24 | 7 | 66 | 1 | 1 |
| Low | Dopamine | 2.922 | 19 | 22 | 2 | 26 | 1 | 61 | 1 | 1 |
| Low | Dopamine | 2.644 | 20 | 15 | 3 | 22 | 3 | 57 | 1 | 1 |
| Low | Dopamine | 3.029 | 21 | 23 | 4 | NA | NA | NA | 1 | NA |
| Low | Dopamine | 2.699 | 22 | 15 | 3 | 22 | 2 | 57 | 1 | 1 |
| Low | Dopamine | 2.777 | 23 | 15 | 2 | 22 | 2 | 61 | 1 | 1 |
| Low | Dopamine | 2.610 | 24 | 15 | 4 | 22 | 2 | 109 | 1 | 1 |
| Low | Dopamine | 2.582 | 25 | 15 | 2 | 22 | 3 | 61 | 1 | 1 |
| Low | Dopamine | 2.878 | 26 | 22 | 3 | 32 | 1 | 61 | 1 | 1 |
| Low | Dopamine | 2.746 | 27 | 21 | 1 | 28 | 1 | 58 | 1 | 1 |
| Low | Dopamine | 2.836 | 28 | 22 | 3 | NA | NA | 57 | 1 | 0 |
| Low | Dopamine | 2.569 | 29 | 15 | 1 | 22 | 3 | 73 | 1 | 1 |
| Low | Dopamine | NA | 30 | NA | NA | NA | NA | NA | NA | NA |
| High | Bupropion | 2.447 | 1 | 7 | 4 | 9 | 8 | 71 | 1 | 1 |
| High | Bupropion | 2.558 | 2 | 7 | 6 | 10 | 6 | 97 | 1 | 1 |
| High | Bupropion | 2.406 | 3 | 7 | 3 | 10 | 8 | 72 | 1 | 1 |
| High | Bupropion | 2.467 | 4 | 7 | 6 | 9 | 7 | 65 | 1 | 1 |
| High | Bupropion | 2.383 | 5 | 7 | 6 | 10 | 7 | 115 | 1 | 1 |
| High | Bupropion | 2.478 | 6 | 7 | 6 | 9 | 9 | 62 | 1 | 1 |
| High | Bupropion | NA | 7 | NA | NA | NA | NA | NA | NA | NA |
| High | Bupropion | 2.472 | 8 | 7 | 4 | 10 | 8 | 74 | 1 | 1 |
| High | Bupropion | 2.752 | 9 | 8 | 6 | 11 | 4 | 90 | 1 | 1 |
| High | Bupropion | 2.548 | 10 | 7 | 4 | 9 | 6 | 67 | 1 | 1 |
| High | Bupropion | 2.555 | 11 | 7 | 7 | 10 | 5 | 76 | 1 | 1 |
| High | Bupropion | 2.452 | 12 | 7 | 6 | 9 | 9 | 57 | 1 | 1 |
| High | Bupropion | 2.498 | 13 | 7 | 6 | 10 | 6 | 62 | 1 | 1 |
| High | Bupropion | 2.544 | 14 | 7 | 4 | 9 | 7 | 83 | 1 | 1 |
| High | Bupropion | 2.476 | 15 | 7 | 5 | 9 | 8 | 62 | 1 | 1 |
| High | Bupropion | 2.442 | 16 | 7 | 6 | 9 | 6 | 63 | 1 | 1 |
| High | Bupropion | 2.582 | 17 | 8 | 7 | 11 | 4 | 63 | 1 | 1 |
| High | Bupropion | 2.439 | 18 | 7 | 4 | 9 | 7 | 78 | 1 | 1 |
| High | Bupropion | 2.500 | 19 | 7 | 4 | 10 | 5 | 80 | 1 | 1 |
| High | Bupropion | 2.594 | 20 | 7 | 8 | 9 | 8 | 65 | 1 | 1 |
| High | Bupropion | 2.463 | 21 | 7 | 5 | 10 | 7 | 44 | 1 | 1 |
| High | Bupropion | 2.665 | 22 | 9 | 6 | 12 | 1 | 75 | 1 | 1 |
| High | Bupropion | 2.588 | 23 | 8 | 6 | 11 | 4 | 95 | 1 | 1 |
| High | Bupropion | 2.576 | 24 | 7 | 6 | 9 | 6 | 75 | 1 | 1 |
| High | Bupropion | 2.486 | 25 | 7 | 3 | 10 | 6 | 58 | 1 | 1 |
| High | Bupropion | 2.440 | 26 | 7 | 7 | 10 | 5 | 57 | 1 | 1 |
| High | Bupropion | 2.707 | 27 | 9 | 6 | 12 | 1 | 72 | 1 | 1 |
| High | Bupropion | 2.597 | 28 | 7 | 8 | 10 | 9 | 78 | 1 | 1 |
| High | Bupropion | 2.441 | 29 | 7 | 5 | 9 | 6 | 75 | 1 | 1 |
| High | Bupropion | 2.483 | 30 | 7 | 8 | 10 | 9 | 62 | 1 | 1 |
| Low | Bupropion | 2.743 | 1 | 16 | 5 | 29 | 1 | 58 | 1 | 1 |
| Low | Bupropion | 2.786 | 2 | 16 | 6 | 28 | 2 | 93 | 1 | 1 |
| Low | Bupropion | 2.779 | 3 | 17 | 6 | 27 | NA | 131 | 1 | 1 |
| Low | Bupropion | 2.760 | 4 | 16 | 6 | 44 | 1 | 97 | 1 | 1 |
| Low | Bupropion | 2.613 | 5 | 16 | 4 | NA | NA | 58 | 1 | 0 |
| Low | Bupropion | 2.802 | 6 | 16 | 7 | 25 | 1 | 58 | 1 | 1 |
| Low | Bupropion | NA | 7 | NA | NA | NA | NA | NA | NA | NA |
| Low | Bupropion | 2.748 | 8 | 16 | 5 | 51 | 2 | 87 | 1 | 1 |
| Low | Bupropion | 2.818 | 9 | 15 | 7 | 51 | 4 | 58 | 1 | 1 |
| Low | Bupropion | 2.654 | 10 | 16 | 5 | 51 | 2 | 85 | 1 | 1 |
| Low | Bupropion | 2.744 | 11 | 17 | 5 | 25 | 1 | 58 | 1 | 1 |
| Low | Bupropion | 2.755 | 12 | 16 | 4 | 55 | 2 | 104 | 1 | 1 |
| Low | Bupropion | 2.718 | 13 | 17 | 7 | NA | NA | 115 | 1 | 0 |
| Low | Bupropion | 2.703 | 14 | 16 | 5 | 51 | 3 | 88 | 1 | 1 |
| Low | Bupropion | 2.805 | 15 | 16 | 6 | 25 | 1 | 100 | 1 | 1 |
| Low | Bupropion | 2.848 | 16 | 17 | 6 | 25 | 1 | 101 | 1 | 1 |
| Low | Bupropion | 2.590 | 17 | 14 | 1 | 17 | 4 | 110 | 1 | 1 |
| Low | Bupropion | 2.761 | 18 | 17 | 4 | 21 | 1 | 88 | 1 | 1 |
| Low | Bupropion | 2.756 | 19 | 15 | 4 | 47 | NA | 107 | 1 | 0 |
| Low | Bupropion | NA | 20 | NA | NA | NA | NA | NA | NA | NA |
| Low | Bupropion | 2.891 | 21 | 25 | 1 | 32 | 1 | 109 | 1 | 1 |
| Low | Bupropion | 3.070 | 22 | 39 | 2 | 43 | 3 | 57 | 1 | 1 |
| Low | Bupropion | 2.676 | 23 | 17 | 1 | 32 | 1 | 95 | 1 | 1 |
| Low | Bupropion | 2.628 | 24 | 18 | 1 | 36 | 1 | 88 | 1 | 1 |
| Low | Bupropion | 3.063 | 25 | 32 | 1 | 35 | 3 | 84 | 1 | 1 |
| Low | Bupropion | 3.071 | 26 | 35 | 1 | 39 | 3 | 91 | 1 | 1 |
| Low | Bupropion | 2.900 | 27 | 39 | 2 | 47 | 1 | 89 | 1 | 1 |
| Low | Bupropion | 2.620 | 28 | 13 | NA | NA | NA | 121 | 1 | NA |
| Low | Bupropion | 2.878 | 29 | 36 | 1 | 44 | 3 | 86 | 1 | 1 |
| Low | Bupropion | 2.784 | 30 | 25 | 1 | 32 | 2 | 113 | 1 | 1 |

**Table A9. Dataset for offspring traits. Replicate id corresponds to mother id.**

| **Food ration** | **Treatment** | **Clutch id** | **Replicate id** | **Offspring size (mm)** |
| --- | --- | --- | --- | --- |
| High | Control | 1 | 1 | 0.744 |
| High | Control | 1 | 1 | 0.853 |
| High | Control | 1 | 1 | 0.818 |
| High | Control | 2 | 1 | 0.801 |
| High | Control | 2 | 1 | 0.86 |
| High | Control | 2 | 1 | 0.821 |
| High | Control | 1 | 2 | 0.754 |
| High | Control | 1 | 2 | 0.735 |
| High | Control | 1 | 2 | 0.738 |
| High | Control | 1 | 3 | 0.719 |
| High | Control | 1 | 3 | 0.767 |
| High | Control | 1 | 3 | 0.71 |
| High | Control | 1 | 4 | 0.746 |
| High | Control | 1 | 4 | 0.671 |
| High | Control | 1 | 4 | 0.784 |
| High | Control | 1 | 5 | 0.751 |
| High | Control | 2 | 5 | 0.758 |
| High | Control | 2 | 5 | 0.748 |
| High | Control | 2 | 5 | 0.771 |
| High | Control | 1 | 6 | 0.792 |
| High | Control | 1 | 6 | 0.82 |
| High | Control | 1 | 6 | 0.799 |
| High | Control | 1 | 7 | 0.774 |
| High | Control | 1 | 7 | 0.823 |
| High | Control | 1 | 8 | 0.745 |
| High | Control | 1 | 8 | 0.832 |
| High | Control | 1 | 8 | 0.806 |
| High | Control | 1 | 9 | 0.848 |
| High | Control | 1 | 9 | 0.858 |
| High | Control | 1 | 9 | 0.842 |
| High | Control | 2 | 9 | 0.871 |
| High | Control | 2 | 9 | 0.903 |
| High | Control | 2 | 9 | 0.925 |
| High | Control | 1 | 10 | 0.795 |
| High | Control | 1 | 10 | 0.802 |
| High | Control | 1 | 11 | 0.739 |
| High | Control | 1 | 11 | 0.76 |
| High | Control | 1 | 11 | 0.756 |
| High | Control | 1 | 12 | 0.771 |
| High | Control | 1 | 12 | 0.762 |
| High | Control | 1 | 12 | 0.832 |
| High | Control | 1 | 13 | 0.839 |
| High | Control | 1 | 13 | 0.834 |
| High | Control | 1 | 14 | 0.775 |
| High | Control | 1 | 14 | 0.821 |
| High | Control | 1 | 14 | 0.685 |
| High | Control | 2 | 14 | 0.861 |
| High | Control | 2 | 14 | 0.81 |
| High | Control | 2 | 14 | 0.819 |
| High | Control | 1 | 15 | 0.787 |
| High | Control | 1 | 15 | 0.81 |
| High | Control | 1 | 15 | 0.821 |
| High | Control | 1 | 16 | 0.708 |
| High | Control | 1 | 16 | 0.729 |
| High | Control | 1 | 16 | 0.669 |
| High | Control | 1 | 17 | 0.795 |
| High | Control | 1 | 17 | 0.646 |
| High | Control | 1 | 18 | 0.681 |
| High | Control | 1 | 18 | 0.732 |
| High | Control | 1 | 18 | 0.68 |
| High | Control | 2 | 18 | 0.817 |
| High | Control | 2 | 18 | 0.844 |
| High | Control | 2 | 18 | 0.871 |
| High | Control | 1 | 19 | 0.68 |
| High | Control | 2 | 19 | 1.013 |
| High | Control | 2 | 19 | 1.013 |
| High | Control | 2 | 19 | 0.955 |
| High | Control | 1 | 20 | 0.916 |
| High | Control | 1 | 20 | 0.949 |
| High | Control | 1 | 20 | 0.985 |
| High | Control | 1 | 21 | 0.952 |
| High | Control | 1 | 21 | 0.874 |
| High | Control | 1 | 21 | 0.976 |
| High | Control | 1 | 22 | 0.982 |
| High | Control | 1 | 22 | 0.981 |
| High | Control | 1 | 22 | 0.916 |
| High | Control | 2 | 22 | 1.108 |
| High | Control | 2 | 22 | 1.087 |
| High | Control | 2 | 22 | 1.118 |
| High | Control | 1 | 23 | 0.73 |
| High | Control | 1 | 23 | 0.746 |
| High | Control | 1 | 23 | 0.831 |
| High | Control | 2 | 23 | 1.042 |
| High | Control | 2 | 23 | 0.877 |
| High | Control | 2 | 23 | 0.882 |
| High | Control | 1 | 24 | 0.749 |
| High | Control | 1 | 24 | 0.733 |
| High | Control | 1 | 24 | 0.743 |
| High | Control | 1 | 25 | 0.773 |
| High | Control | 1 | 25 | 0.838 |
| High | Control | 1 | 25 | 0.745 |
| High | Control | 1 | 27 | 0.745 |
| High | Control | 1 | 27 | 0.734 |
| High | Control | 1 | 27 | 0.736 |
| High | Control | 1 | 28 | 0.776 |
| High | Control | 1 | 28 | 0.789 |
| High | Control | 1 | 28 | 0.778 |
| High | Control | 1 | 29 | 0.739 |
| High | Control | 1 | 29 | 0.65 |
| High | Control | 1 | 29 | 0.721 |
| High | Control | 2 | 29 | 0.819 |
| High | Control | 2 | 29 | 0.843 |
| High | Control | 2 | 29 | 0.787 |
| High | Control | 1 | 30 | 0.766 |
| High | Control | 1 | 30 | 0.741 |
| High | Control | 1 | 30 | 0.733 |
| Low | Control | 1 | 1 | 0.967 |
| Low | Control | 1 | 1 | 0.949 |
| Low | Control | 2 | 1 | 0.982 |
| Low | Control | 2 | 1 | 0.977 |
| Low | Control | 2 | 1 | 0.981 |
| Low | Control | 1 | 2 | 0.867 |
| Low | Control | 1 | 2 | 0.857 |
| Low | Control | 2 | 2 | 0.875 |
| Low | Control | 2 | 2 | 0.885 |
| Low | Control | 1 | 3 | 0.912 |
| Low | Control | 2 | 3 | 1.103 |
| Low | Control | 1 | 4 | 0.859 |
| Low | Control | 2 | 4 | 0.788 |
| Low | Control | 2 | 4 | 0.798 |
| Low | Control | 1 | 5 | 0.82 |
| Low | Control | 2 | 5 | 0.755 |
| Low | Control | 1 | 6 | 0.973 |
| Low | Control | 2 | 6 | 0.969 |
| Low | Control | 1 | 7 | 0.939 |
| Low | Control | 2 | 7 | 1.002 |
| Low | Control | 2 | 7 | 1.011 |
| Low | Control | 1 | 8 | 0.915 |
| Low | Control | 1 | 9 | 0.908 |
| Low | Control | 2 | 9 | 0.992 |
| Low | Control | 1 | 10 | 0.777 |
| Low | Control | 1 | 10 | 0.734 |
| Low | Control | 2 | 10 | 0.939 |
| Low | Control | 2 | 10 | 0.997 |
| Low | Control | 1 | 11 | 1.016 |
| Low | Control | 1 | 11 | 0.977 |
| Low | Control | 1 | 12 | 0.948 |
| Low | Control | 1 | 12 | 0.881 |
| Low | Control | 1 | 13 | 0.803 |
| Low | Control | 2 | 13 | 0.976 |
| Low | Control | 1 | 14 | 0.941 |
| Low | Control | 2 | 14 | 1.006 |
| Low | Control | 2 | 14 | 0.973 |
| Low | Control | 1 | 15 | 0.975 |
| Low | Control | 1 | 15 | 0.984 |
| Low | Control | 1 | 15 | 0.955 |
| Low | Control | 2 | 15 | 0.987 |
| Low | Control | 2 | 15 | 0.962 |
| Low | Control | 2 | 15 | 0.953 |
| Low | Control | 1 | 16 | 0.972 |
| Low | Control | 1 | 16 | 0.977 |
| Low | Control | 2 | 16 | 0.926 |
| Low | Control | 2 | 16 | 0.939 |
| Low | Control | 1 | 17 | 0.955 |
| Low | Control | 1 | 17 | 0.939 |
| Low | Control | 1 | 17 | 0.974 |
| Low | Control | 2 | 17 | 0.996 |
| Low | Control | 2 | 17 | 0.993 |
| Low | Control | 2 | 17 | 0.99 |
| Low | Control | 1 | 18 | 1.05 |
| Low | Control | 1 | 20 | 0.94 |
| Low | Control | 1 | 20 | 0.902 |
| Low | Control | 1 | 20 | 0.931 |
| Low | Control | 2 | 20 | 1.012 |
| Low | Control | 2 | 20 | 1.045 |
| Low | Control | 1 | 21 | 0.944 |
| Low | Control | 1 | 21 | 0.923 |
| Low | Control | 1 | 22 | 0.866 |
| Low | Control | 1 | 22 | 0.898 |
| Low | Control | 1 | 22 | 0.874 |
| Low | Control | 2 | 22 | 0.928 |
| Low | Control | 2 | 22 | 0.919 |
| Low | Control | 1 | 23 | 0.911 |
| Low | Control | 1 | 23 | 0.937 |
| Low | Control | 2 | 23 | 0.975 |
| Low | Control | 2 | 23 | 0.949 |
| Low | Control | 1 | 24 | 1.012 |
| Low | Control | 1 | 24 | 0.979 |
| Low | Control | 1 | 26 | 1.005 |
| Low | Control | 2 | 26 | 1.016 |
| Low | Control | 1 | 27 | 1.007 |
| Low | Control | 1 | 27 | 1.161 |
| Low | Control | 2 | 27 | 0.994 |
| Low | Control | 2 | 27 | 1.027 |
| Low | Control | 2 | 27 | 1.004 |
| Low | Control | 1 | 28 | 0.933 |
| Low | Control | 1 | 28 | 0.974 |
| Low | Control | 1 | 29 | 0.976 |
| Low | Control | 1 | 29 | 0.981 |
| High | Dopamine | 1 | 2 | 0.837 |
| High | Dopamine | 1 | 2 | 0.823 |
| High | Dopamine | 1 | 2 | 0.816 |
| High | Dopamine | 1 | 4 | 0.669 |
| High | Dopamine | 1 | 4 | 0.631 |
| High | Dopamine | 1 | 4 | 0.652 |
| High | Dopamine | 1 | 6 | 0.806 |
| High | Dopamine | 1 | 6 | 0.804 |
| High | Dopamine | 1 | 6 | 0.775 |
| High | Dopamine | 1 | 7 | 0.719 |
| High | Dopamine | 1 | 7 | 0.688 |
| High | Dopamine | 1 | 7 | 0.692 |
| High | Dopamine | 1 | 8 | 0.649 |
| High | Dopamine | 1 | 8 | 0.684 |
| High | Dopamine | 1 | 8 | 0.616 |
| High | Dopamine | 1 | 9 | 0.674 |
| High | Dopamine | 1 | 9 | 0.633 |
| High | Dopamine | 1 | 9 | 0.495 |
| High | Dopamine | 1 | 10 | 0.652 |
| High | Dopamine | 1 | 10 | 0.679 |
| High | Dopamine | 1 | 10 | 0.723 |
| High | Dopamine | 1 | 12 | 0.732 |
| High | Dopamine | 1 | 12 | 0.663 |
| High | Dopamine | 1 | 12 | 0.756 |
| High | Dopamine | 1 | 13 | 0.785 |
| High | Dopamine | 1 | 13 | 0.799 |
| High | Dopamine | 1 | 13 | 0.79 |
| High | Dopamine | 2 | 14 | 0.838 |
| High | Dopamine | 2 | 14 | 0.869 |
| High | Dopamine | 2 | 14 | 0.848 |
| High | Dopamine | 1 | 15 | 0.753 |
| High | Dopamine | 1 | 15 | 0.711 |
| High | Dopamine | 1 | 16 | 0.777 |
| High | Dopamine | 1 | 16 | 0.747 |
| High | Dopamine | 1 | 16 | 0.764 |
| High | Dopamine | 1 | 17 | 0.797 |
| High | Dopamine | 1 | 17 | 0.85 |
| High | Dopamine | 1 | 17 | 0.836 |
| High | Dopamine | 1 | 18 | 0.78 |
| High | Dopamine | 1 | 18 | 0.825 |
| High | Dopamine | 1 | 18 | 0.738 |
| High | Dopamine | 1 | 19 | 0.788 |
| High | Dopamine | 1 | 19 | 0.751 |
| High | Dopamine | 1 | 19 | 0.645 |
| High | Dopamine | 1 | 20 | 0.774 |
| High | Dopamine | 1 | 20 | 0.759 |
| High | Dopamine | 1 | 20 | 0.741 |
| High | Dopamine | 1 | 21 | 0.785 |
| High | Dopamine | 1 | 21 | 0.762 |
| High | Dopamine | 1 | 21 | 0.773 |
| High | Dopamine | 1 | 22 | 0.696 |
| High | Dopamine | 1 | 22 | 0.619 |
| High | Dopamine | 1 | 22 | 0.734 |
| High | Dopamine | 1 | 23 | 0.669 |
| High | Dopamine | 1 | 23 | 0.786 |
| High | Dopamine | 1 | 23 | 0.787 |
| High | Dopamine | 1 | 24 | 0.708 |
| High | Dopamine | 1 | 24 | 0.723 |
| High | Dopamine | 1 | 24 | 0.736 |
| High | Dopamine | 1 | 25 | 0.79 |
| High | Dopamine | 1 | 25 | 0.759 |
| High | Dopamine | 1 | 26 | 0.767 |
| High | Dopamine | 1 | 26 | 0.713 |
| High | Dopamine | 1 | 26 | 0.791 |
| High | Dopamine | 1 | 27 | 0.752 |
| High | Dopamine | 1 | 27 | 0.757 |
| High | Dopamine | 1 | 27 | 0.76 |
| High | Dopamine | 1 | 28 | 0.69 |
| High | Dopamine | 1 | 28 | 0.709 |
| High | Dopamine | 1 | 28 | 0.729 |
| High | Dopamine | 1 | 29 | 0.828 |
| High | Dopamine | 1 | 29 | 0.876 |
| High | Dopamine | 1 | 29 | 0.859 |
| High | Dopamine | 1 | 30 | 0.822 |
| High | Dopamine | 1 | 30 | 0.866 |
| High | Dopamine | 1 | 30 | 0.853 |
| Low | Dopamine | 1 | 1 | 0.896 |
| Low | Dopamine | 2 | 1 | 0.991 |
| Low | Dopamine | 1 | 2 | 0.972 |
| Low | Dopamine | 1 | 2 | 0.977 |
| Low | Dopamine | 1 | 3 | 0.786 |
| Low | Dopamine | 1 | 3 | 0.933 |
| Low | Dopamine | 1 | 3 | 0.899 |
| Low | Dopamine | 2 | 3 | 1.002 |
| Low | Dopamine | 1 | 4 | 0.921 |
| Low | Dopamine | 1 | 4 | 0.945 |
| Low | Dopamine | 2 | 4 | 0.984 |
| Low | Dopamine | 2 | 4 | 0.958 |
| Low | Dopamine | 1 | 5 | 0.945 |
| Low | Dopamine | 1 | 5 | 0.978 |
| Low | Dopamine | 2 | 5 | 0.841 |
| Low | Dopamine | 2 | 5 | 0.842 |
| Low | Dopamine | 2 | 5 | 0.818 |
| Low | Dopamine | 1 | 6 | 0.947 |
| Low | Dopamine | 1 | 6 | 0.943 |
| Low | Dopamine | 2 | 6 | 0.866 |
| Low | Dopamine | 1 | 7 | 0.897 |
| Low | Dopamine | 1 | 7 | 0.913 |
| Low | Dopamine | 2 | 7 | 0.912 |
| Low | Dopamine | 2 | 7 | 0.866 |
| Low | Dopamine | 2 | 7 | 0.941 |
| Low | Dopamine | 1 | 8 | 0.907 |
| Low | Dopamine | 1 | 8 | 0.974 |
| Low | Dopamine | 1 | 9 | 0.976 |
| Low | Dopamine | 2 | 9 | 0.782 |
| Low | Dopamine | 2 | 9 | 0.835 |
| Low | Dopamine | 2 | 9 | 0.907 |
| Low | Dopamine | 1 | 10 | 0.981 |
| Low | Dopamine | 1 | 10 | 1.13 |
| Low | Dopamine | 2 | 10 | 0.901 |
| Low | Dopamine | 2 | 10 | 0.858 |
| Low | Dopamine | 1 | 11 | 0.843 |
| Low | Dopamine | 1 | 11 | 0.813 |
| Low | Dopamine | 1 | 11 | 0.786 |
| Low | Dopamine | 2 | 11 | 0.913 |
| Low | Dopamine | 2 | 11 | 0.937 |
| Low | Dopamine | 1 | 12 | 0.878 |
| Low | Dopamine | 1 | 12 | 0.903 |
| Low | Dopamine | 2 | 12 | 0.853 |
| Low | Dopamine | 2 | 12 | 0.876 |
| Low | Dopamine | 2 | 12 | 0.836 |
| Low | Dopamine | 1 | 13 | 0.888 |
| Low | Dopamine | 1 | 13 | 0.843 |
| Low | Dopamine | 1 | 13 | 0.902 |
| Low | Dopamine | 2 | 13 | 0.852 |
| Low | Dopamine | 2 | 13 | 0.844 |
| Low | Dopamine | 2 | 13 | 0.899 |
| Low | Dopamine | 1 | 14 | 0.944 |
| Low | Dopamine | 1 | 14 | 0.926 |
| Low | Dopamine | 2 | 14 | 0.907 |
| Low | Dopamine | 2 | 14 | 0.874 |
| Low | Dopamine | 2 | 14 | 0.859 |
| Low | Dopamine | 1 | 15 | 0.848 |
| Low | Dopamine | 1 | 15 | 0.872 |
| Low | Dopamine | 1 | 15 | 0.832 |
| Low | Dopamine | 2 | 15 | 0.908 |
| Low | Dopamine | 2 | 15 | 0.95 |
| Low | Dopamine | 2 | 15 | 0.956 |
| Low | Dopamine | 1 | 16 | 0.864 |
| Low | Dopamine | 1 | 16 | 0.843 |
| Low | Dopamine | 1 | 16 | 0.877 |
| Low | Dopamine | 1 | 17 | 0.922 |
| Low | Dopamine | 1 | 17 | 0.892 |
| Low | Dopamine | 1 | 17 | 0.889 |
| Low | Dopamine | 2 | 17 | 0.804 |
| Low | Dopamine | 2 | 17 | 0.902 |
| Low | Dopamine | 2 | 17 | 0.948 |
| Low | Dopamine | 1 | 18 | 0.968 |
| Low | Dopamine | 1 | 18 | 0.932 |
| Low | Dopamine | 2 | 18 | 0.855 |
| Low | Dopamine | 2 | 18 | 0.863 |
| Low | Dopamine | 2 | 18 | 0.811 |
| Low | Dopamine | 1 | 19 | 0.944 |
| Low | Dopamine | 1 | 19 | 0.972 |
| Low | Dopamine | 1 | 20 | 0.822 |
| Low | Dopamine | 1 | 20 | 0.77 |
| Low | Dopamine | 1 | 20 | 0.807 |
| Low | Dopamine | 2 | 20 | 0.86 |
| Low | Dopamine | 2 | 20 | 0.83 |
| Low | Dopamine | 2 | 20 | 0.907 |
| Low | Dopamine | 1 | 21 | 0.817 |
| Low | Dopamine | 1 | 21 | 0.788 |
| Low | Dopamine | 1 | 21 | 0.784 |
| Low | Dopamine | 1 | 22 | 0.905 |
| Low | Dopamine | 1 | 22 | 0.877 |
| Low | Dopamine | 1 | 22 | 0.877 |
| Low | Dopamine | 2 | 22 | 0.865 |
| Low | Dopamine | 2 | 22 | 0.878 |
| Low | Dopamine | 1 | 23 | 0.764 |
| Low | Dopamine | 1 | 23 | 0.726 |
| Low | Dopamine | 1 | 23 | 0.726 |
| Low | Dopamine | 2 | 23 | 0.866 |
| Low | Dopamine | 2 | 23 | 0.871 |
| Low | Dopamine | 1 | 24 | 0.849 |
| Low | Dopamine | 1 | 24 | 0.856 |
| Low | Dopamine | 1 | 24 | 0.861 |
| Low | Dopamine | 2 | 24 | 0.935 |
| Low | Dopamine | 2 | 24 | 0.967 |
| Low | Dopamine | 1 | 25 | 0.749 |
| Low | Dopamine | 2 | 25 | 0.831 |
| Low | Dopamine | 2 | 25 | 0.872 |
| Low | Dopamine | 2 | 25 | 0.875 |
| Low | Dopamine | 1 | 26 | 0.924 |
| Low | Dopamine | 1 | 26 | 0.872 |
| Low | Dopamine | 1 | 26 | 0.905 |
| Low | Dopamine | 2 | 26 | 0.912 |
| Low | Dopamine | 1 | 27 | 0.845 |
| Low | Dopamine | 1 | 28 | 0.86 |
| Low | Dopamine | 1 | 28 | 0.876 |
| Low | Dopamine | 1 | 28 | 0.889 |
| Low | Dopamine | 1 | 29 | 0.955 |
| Low | Dopamine | 1 | 29 | 0.652 |
| Low | Dopamine | 2 | 29 | 0.844 |
| Low | Dopamine | 2 | 29 | 0.886 |
| Low | Dopamine | 2 | 29 | 0.841 |
| High | Bupropion | 1 | 1 | 0.732 |
| High | Bupropion | 1 | 1 | 0.652 |
| High | Bupropion | 1 | 1 | 0.642 |
| High | Bupropion | 1 | 2 | 0.742 |
| High | Bupropion | 1 | 2 | 0.62 |
| High | Bupropion | 1 | 2 | 0.747 |
| High | Bupropion | 1 | 3 | 0.616 |
| High | Bupropion | 1 | 3 | 0.58 |
| High | Bupropion | 2 | 3 | 0.805 |
| High | Bupropion | 2 | 3 | 0.776 |
| High | Bupropion | 2 | 3 | 0.776 |
| High | Bupropion | 1 | 4 | 0.768 |
| High | Bupropion | 1 | 4 | 0.727 |
| High | Bupropion | 1 | 4 | 0.706 |
| High | Bupropion | 1 | 5 | 0.716 |
| High | Bupropion | 1 | 5 | 0.575 |
| High | Bupropion | 1 | 5 | 0.695 |
| High | Bupropion | 1 | 6 | 0.665 |
| High | Bupropion | 1 | 6 | 0.704 |
| High | Bupropion | 1 | 6 | 0.687 |
| High | Bupropion | 1 | 8 | 0.502 |
| High | Bupropion | 1 | 8 | 0.585 |
| High | Bupropion | 1 | 8 | 0.608 |
| High | Bupropion | 2 | 8 | 0.863 |
| High | Bupropion | 2 | 8 | 0.887 |
| High | Bupropion | 2 | 8 | 0.837 |
| High | Bupropion | 1 | 9 | 0.729 |
| High | Bupropion | 1 | 9 | 0.826 |
| High | Bupropion | 1 | 9 | 0.7 |
| High | Bupropion | 1 | 10 | 0.68 |
| High | Bupropion | 1 | 10 | 0.576 |
| High | Bupropion | 1 | 10 | 0.628 |
| High | Bupropion | 1 | 11 | 0.728 |
| High | Bupropion | 1 | 11 | 0.762 |
| High | Bupropion | 1 | 11 | 0.725 |
| High | Bupropion | 1 | 12 | 0.638 |
| High | Bupropion | 1 | 12 | 0.595 |
| High | Bupropion | 1 | 12 | 0.652 |
| High | Bupropion | 1 | 13 | 0.732 |
| High | Bupropion | 1 | 13 | 0.738 |
| High | Bupropion | 1 | 13 | 0.727 |
| High | Bupropion | 1 | 14 | 0.68 |
| High | Bupropion | 1 | 14 | 0.743 |
| High | Bupropion | 1 | 14 | 0.694 |
| High | Bupropion | 1 | 15 | 0.711 |
| High | Bupropion | 1 | 15 | 0.775 |
| High | Bupropion | 1 | 15 | 0.658 |
| High | Bupropion | 1 | 16 | 0.616 |
| High | Bupropion | 1 | 16 | 0.565 |
| High | Bupropion | 1 | 16 | 0.661 |
| High | Bupropion | 1 | 17 | 0.741 |
| High | Bupropion | 1 | 17 | 0.818 |
| High | Bupropion | 1 | 17 | 0.785 |
| High | Bupropion | 1 | 18 | 0.722 |
| High | Bupropion | 1 | 18 | 0.6 |
| High | Bupropion | 1 | 18 | 0.726 |
| High | Bupropion | 1 | 20 | 0.641 |
| High | Bupropion | 1 | 20 | 0.718 |
| High | Bupropion | 1 | 20 | 0.705 |
| High | Bupropion | 1 | 21 | 0.708 |
| High | Bupropion | 1 | 21 | 0.686 |
| High | Bupropion | 1 | 21 | 0.641 |
| High | Bupropion | 1 | 22 | 0.826 |
| High | Bupropion | 1 | 22 | 0.823 |
| High | Bupropion | 1 | 22 | 0.796 |
| High | Bupropion | 1 | 23 | 0.8 |
| High | Bupropion | 1 | 23 | 0.832 |
| High | Bupropion | 1 | 24 | 0.705 |
| High | Bupropion | 1 | 24 | 0.747 |
| High | Bupropion | 1 | 24 | 0.755 |
| High | Bupropion | 1 | 25 | 0.703 |
| High | Bupropion | 1 | 25 | 0.765 |
| High | Bupropion | 1 | 25 | 0.766 |
| High | Bupropion | 1 | 26 | 0.716 |
| High | Bupropion | 1 | 26 | 0.593 |
| High | Bupropion | 1 | 26 | 0.698 |
| High | Bupropion | 1 | 27 | 0.898 |
| High | Bupropion | 1 | 27 | 0.897 |
| High | Bupropion | 1 | 27 | 0.843 |
| High | Bupropion | 1 | 28 | 0.68 |
| High | Bupropion | 1 | 28 | 0.736 |
| High | Bupropion | 1 | 28 | 0.76 |
| High | Bupropion | 1 | 29 | 0.584 |
| High | Bupropion | 1 | 29 | 0.525 |
| High | Bupropion | 1 | 29 | 0.567 |
| High | Bupropion | 1 | 30 | 0.751 |
| High | Bupropion | 1 | 30 | 0.644 |
| High | Bupropion | 1 | 30 | 0.742 |
| High | Bupropion | 2 | 30 | 0.838 |
| High | Bupropion | 2 | 30 | 0.774 |
| High | Bupropion | 2 | 30 | 0.852 |
| Low | Bupropion | 1 | 1 | 0.844 |
| Low | Bupropion | 1 | 1 | 0.867 |
| Low | Bupropion | 1 | 1 | 0.875 |
| Low | Bupropion | 2 | 1 | 0.93 |
| Low | Bupropion | 2 | 1 | 0.976 |
| Low | Bupropion | 1 | 2 | 0.757 |
| Low | Bupropion | 1 | 2 | 0.779 |
| Low | Bupropion | 1 | 2 | 0.771 |
| Low | Bupropion | 2 | 2 | 0.992 |
| Low | Bupropion | 2 | 2 | 0.985 |
| Low | Bupropion | 1 | 3 | 0.84 |
| Low | Bupropion | 1 | 3 | 0.835 |
| Low | Bupropion | 1 | 3 | 0.888 |
| Low | Bupropion | 1 | 4 | 0.709 |
| Low | Bupropion | 1 | 4 | 0.645 |
| Low | Bupropion | 2 | 4 | 0.991 |
| Low | Bupropion | 1 | 5 | 0.895 |
| Low | Bupropion | 1 | 5 | 0.869 |
| Low | Bupropion | 1 | 6 | 0.848 |
| Low | Bupropion | 1 | 6 | 0.865 |
| Low | Bupropion | 1 | 6 | 0.838 |
| Low | Bupropion | 2 | 6 | 0.862 |
| Low | Bupropion | 1 | 8 | 0.848 |
| Low | Bupropion | 1 | 8 | 0.814 |
| Low | Bupropion | 1 | 8 | 0.835 |
| Low | Bupropion | 2 | 8 | 0.967 |
| Low | Bupropion | 2 | 8 | 0.947 |
| Low | Bupropion | 1 | 9 | 0.777 |
| Low | Bupropion | 1 | 9 | 0.864 |
| Low | Bupropion | 1 | 9 | 0.822 |
| Low | Bupropion | 2 | 9 | 0.903 |
| Low | Bupropion | 2 | 9 | 0.919 |
| Low | Bupropion | 2 | 9 | 0.894 |
| Low | Bupropion | 1 | 10 | 0.831 |
| Low | Bupropion | 1 | 10 | 0.853 |
| Low | Bupropion | 1 | 10 | 0.859 |
| Low | Bupropion | 2 | 10 | 0.961 |
| Low | Bupropion | 2 | 10 | 0.961 |
| Low | Bupropion | 1 | 11 | 0.769 |
| Low | Bupropion | 1 | 11 | 0.784 |
| Low | Bupropion | 1 | 11 | 0.727 |
| Low | Bupropion | 2 | 11 | 0.96 |
| Low | Bupropion | 1 | 12 | 0.823 |
| Low | Bupropion | 1 | 12 | 0.837 |
| Low | Bupropion | 1 | 12 | 0.882 |
| Low | Bupropion | 2 | 12 | 0.938 |
| Low | Bupropion | 2 | 12 | 0.962 |
| Low | Bupropion | 1 | 13 | 0.817 |
| Low | Bupropion | 1 | 13 | 0.774 |
| Low | Bupropion | 1 | 13 | 0.818 |
| Low | Bupropion | 2 | 13 | 0.959 |
| Low | Bupropion | 1 | 14 | 0.907 |
| Low | Bupropion | 1 | 14 | 0.896 |
| Low | Bupropion | 1 | 14 | 0.92 |
| Low | Bupropion | 2 | 14 | 0.934 |
| Low | Bupropion | 2 | 14 | 0.986 |
| Low | Bupropion | 2 | 14 | 1.003 |
| Low | Bupropion | 1 | 15 | 0.834 |
| Low | Bupropion | 1 | 15 | 0.861 |
| Low | Bupropion | 1 | 15 | 0.855 |
| Low | Bupropion | 2 | 15 | 0.86 |
| Low | Bupropion | 1 | 16 | 0.841 |
| Low | Bupropion | 1 | 16 | 0.826 |
| Low | Bupropion | 1 | 16 | 0.831 |
| Low | Bupropion | 2 | 16 | 0.86 |
| Low | Bupropion | 1 | 17 | 0.889 |
| Low | Bupropion | 2 | 17 | 0.859 |
| Low | Bupropion | 2 | 17 | 0.882 |
| Low | Bupropion | 1 | 18 | 0.54 |
| Low | Bupropion | 1 | 18 | 0.601 |
| Low | Bupropion | 1 | 19 | 0.739 |
| Low | Bupropion | 1 | 19 | 0.736 |
| Low | Bupropion | 1 | 19 | 0.678 |
| Low | Bupropion | 2 | 19 | 0.962 |
| Low | Bupropion | 2 | 19 | 0.916 |
| Low | Bupropion | 2 | 19 | 0.795 |
| Low | Bupropion | 1 | 21 | 0.974 |
| Low | Bupropion | 2 | 21 | 0.738 |
| Low | Bupropion | 1 | 22 | 0.978 |
| Low | Bupropion | 1 | 22 | 0.977 |
| Low | Bupropion | 2 | 22 | 0.98 |
| Low | Bupropion | 1 | 23 | 0.862 |
| Low | Bupropion | 2 | 23 | 0.889 |
| Low | Bupropion | 2 | 24 | 1.003 |
| Low | Bupropion | 1 | 25 | 0.913 |
| Low | Bupropion | 2 | 25 | 0.987 |
| Low | Bupropion | 2 | 25 | 0.905 |
| Low | Bupropion | 2 | 25 | 0.978 |
| Low | Bupropion | 1 | 26 | 0.993 |
| Low | Bupropion | 2 | 26 | 0.904 |
| Low | Bupropion | 2 | 26 | 0.955 |
| Low | Bupropion | 1 | 27 | 0.88 |
| Low | Bupropion | 1 | 27 | 0.881 |
| Low | Bupropion | 2 | 27 | 0.988 |
| Low | Bupropion | 1 | 29 | 0.99 |
| Low | Bupropion | 1 | 30 | 0.849 |
| Low | Bupropion | 2 | 30 | 0.585 |

**Table A10. Dataset for exposure and water quality variables.**

| **Sampling event** | **Treatment** | **Dopamine (mg/L)** | **Bupropion (µg/L)** | **Conductivity (mS/cm)** | **Dissolved oxygen (mg/L)** | **pH** |
| --- | --- | --- | --- | --- | --- | --- |
| 1 | Dopamine | 0.11 | 0 | 2.27 | 9.05 | 8.2 |
| 1 | Bupropion | 0 | 0.32 | 2.32 | 8.98 | 8.25 |
| 1 | Control | 0 | 0 | 2.26 | 8.96 | 8.263 |
| 2 | Dopamine | 0.22 | 0 | NA | NA | NA |
| 2 | Bupropion | 0 | 0.15 | NA | NA | NA |
| 2 | Control | 0 | 0 | NA | NA | NA |
| 3 | Dopamine | 0.08 | 0 | 2.1 | 8.99 | 8.4 |
| 3 | Bupropion | 0 | 0.064 | 2.15 | 8.95 | 8.37 |
| 3 | Control | 0 | 0 | 2.09 | 9.06 | 8.57 |
| 4 | Dopamine | 0.44 | 0 | 2.08 | 8.94 | 8.45 |
| 4 | Bupropion | 0 | 0 | 2.12 | 8.94 | 8.4 |
| 4 | Control | 0 | 0 | 2.08 | 8.92 | 8.6 |
| 5 | Dopamine | NA | 0 | 0.496 | 8.82 | 8.175 |
| 5 | Bupropion | 0 | NA | 0.5 | 8.685 | 8.25 |
| 5 | Control | 0 | 0 | 0.511 | 8.97 | 8.23 |
| 6 | Dopamine | 0 | 0 | 0.498 | 9.15 | 8.43 |
| 6 | Bupropion | 0 | 0.015 | 0.504 | 9.12 | 8.76 |
| 6 | Control | 0 | 0 | 0.498 | 9.3 | 8.85 |
| 7 | Dopamine | 0 | 0 | 0.514 | 8.87 | 8.565 |
| 7 | Bupropion | 0 | 0.031 | 0.515 | 8.9 | 8.488 |
| 7 | Control | 0 | 0 | 0.522 | 8.86 | 8.501 |
| 8 | Dopamine | 1.17 | 0 | 0.524 | 8.72 | 7.74 |
| 8 | Bupropion | 0 | 0.09 | 0.527 | 8.92 | 7.77 |
| 8 | Control | 0 | 0 | 0.527 | 8.95 | 7.9 |
| 9 | Dopamine | NA | 0 | 0.535 | 9.03 | 8.02 |
| 9 | Bupropion | 0 | NA | 0.54 | 9.07 | 8.03 |
| 9 | Control | 0 | 0 | 0.539 | 8.95 | 8.15 |
| 10 | Dopamine | NA | 0 | 0.501 | 9.13 | 8.09 |
| 10 | Bupropion | 0 | NA | 0.507 | 9.15 | 8.04 |
| 10 | Control | 0 | 0 | 0.505 | 9.24 | 8.055 |

**Reference**

1. Asimakopoulos AG, Kannan P, Higgins S, Kannan K. Determination of 89 drugs and other micropollutants in unfiltered wastewater and freshwater by LC-MS/MS: An alternative sample preparation approach. Analytical and Bioanalytical Chemistry. 2017;409:6205-25.
